# Suan-Zao-Ren decoction for insomnia: A protocol for a systematic review and meta-analysis

**DOI:** 10.1097/MD.0000000000021658

**Published:** 2020-08-21

**Authors:** Zhijian Song, Ping Fan, Qi Zhang, Yang Yang, Qinan Zhan, Xueyu Liu, Yurong Xiong

**Affiliations:** a Jiangxi Provincial Hospital of Integrated Traditional Chinese and Western Medicine; b Jiangxi University of Traditional Chinese Medicine, Nanchang, China.

**Keywords:** insomnia, protocol, Suan Zao Ren decoction, systematic review

## Abstract

**Background:**

Chinese herbal medicine has been widely used to relieve insomnia. Among them, Suan-Zao-Ren decoction (SZRD) has a significant effect in alleviating insomnia. The purpose of this systematic review is to evaluate the effectiveness and safety of SZRD in treating insomnia.

**Methods:**

Relevant randomized controlled trials (RCTs) will be searched from the databases of Embase, PubMed, the Cochrane Library, the China National Knowledge Infrastructure, Wanfang Database and Chinese Science and Technology Periodical Database from their inception to July 2020. Two independent reviewers will select studies, collect data, and assess the methodology quality by the Cochrane risk of bias tool. Statistical analysis is processed by RevMan V.5.3 software.

**Results:**

The results of this systematic review will provide an assessment of SZRD treatment of insomnia, and aims to prove the effectiveness and safety of SZRD.

**Conclusion:**

This study will provide a credible Evidence-based for the treatment of Insomnia with SZRD.

## 1 Introduction

Insomnia is a common public health problem, affecting about 40% to 50% of the general population,^[1]^ and its prevalence is increasing.^[2]^ In the United States, it is estimated that one-third of adults are experiencing insomnia.^[3]^ Long-term insomnia seriously affects the quality of life of patients. At the same time, insomnia increases the risk of many health problems, such as cardiovascular disease, hypertension, myocardial infarction chronic heart failure and depression, etc.^[4–6]^ In addition, insomnia also leads to a decline in work performance. In the United States, the estimated annual economic loss due to insomnia is more than $63 billion.^[7]^ Therefore, insomnia puts a heavy burden on individuals and society.^[8,9]^

As far as the treatment of insomnia is concerned, pharmacotherapy is a common method and recommended by clinical guidelines.^[8,10]^ Although the short-term efficacy of pharmacotherapy is obvious, its long-term clinical application is limited by side effects, including dependence, dizziness and gastrointestinal reactions.^[11,12]^ Therefore, it is necessary to explore more safe and effective alternative therapies to treat insomnia. Chinese Herbal Medicine (CHM) is one of the most popular alternative treatments.^[13,14]^ Recent systematic review^[15]^ supports the use of CHM as a whole for insomnia.

Suan-Zao-Ren decoction (SZRD) is a well-known formula, which is composed of five herbs, namely Ziziphi Spinosae Semen (ZSS), Poria (P), Chuanxiong Rhizoma (CR), Anemarrhenae Rhizoma (AR), and Glycyrrhizae Radix Et Rhizoma (GRR). As a representative drug, SZRD has been widely used to treat insomnia and anxiety disorders for thousands of years.^[16,17]^ Modern pharmacological studies have shown that SZRD has anti-convulsant, nourish blood, protect cardiovascular system and enhance immunity.^[18,19]^ Therefore, in modern clinical treatment, SZRD is considered to be a safe, effective, hygienic, convenient, and inexpensive supplemental alternative treatment method.^[20]^

Although the benefits of SZRD in the treatment of insomnia have been widely reported, the effectiveness of SZRD has not been systematically and scientifically evaluated. This study uses the method of evidence-based medicine to analyze and evaluate the RCTs of patients with Insomnia treated by SZRD to clarify the safety and effectiveness of SZRD for insomnia.

## 2 Methods

The protocol of this study has been registered with the Open Science Framework (OSF, https://osf.io/). The registration DOI of this review is 10.17605/OSF.IO/4P6K8. We will refer to the preferred reporting items for the systematic review and meta-analysis (PRISMA) to perform this study.

### 2.1 Inclusion criteria for study selection

#### 2.1.1 Types of studies

RCTs assessing SZRD treatment for Insomnia will be eligible for inclusion. No language and publication status restrictions.

#### 2.1.2 Types of participants

Insomnia patients with definite diagnosis will be included in this systematic review. There are no restrictions on race, age, gender, or nationality.

#### 2.1.3 Types of interventions

##### 2.1.3.1 Experimental interventions

Using SZRD or modified SZRD as the experimental intervention, regardless of the administration type, dose, or intervention duration.

##### 2.1.3.2 Control interventions

The control group will receive one of the following treatment methods: pharmacotherapy, Cognitive-Behavioral Therapy (CBT) or placebo.

#### 2.1.4 Types of outcome measures

##### 2.1.4.1 Primary outcome

Pittsburgh sleep quality index (PSQI) and Clinical efficacy will be accepted as the primary outcomes.

##### 2.1.4.2 Additional outcomes

The safety assessment will be regarded as an additional result.

### 2.2 Search methods for the identification of studies

The following databases will be searched: PubMed, Embase, Cochrane Library, the China National Knowledge Infrastructure, Chinese Science and Technology Periodical Database and Wanfang Database. We will search the databases from the beginning to July 2020. Search terms consist of disease (insomnia OR sleep OR sleepless) and intervention (Suan-Zao-Ren decoction OR Suan-Zao-Ren Tang OR Suan Zao Ren OR SuanZaoRen) and research types (randomized controlled trial, controlled clinical trial, random trials). The PubMed search strategy is shown in Table 1.

**Table 1:**
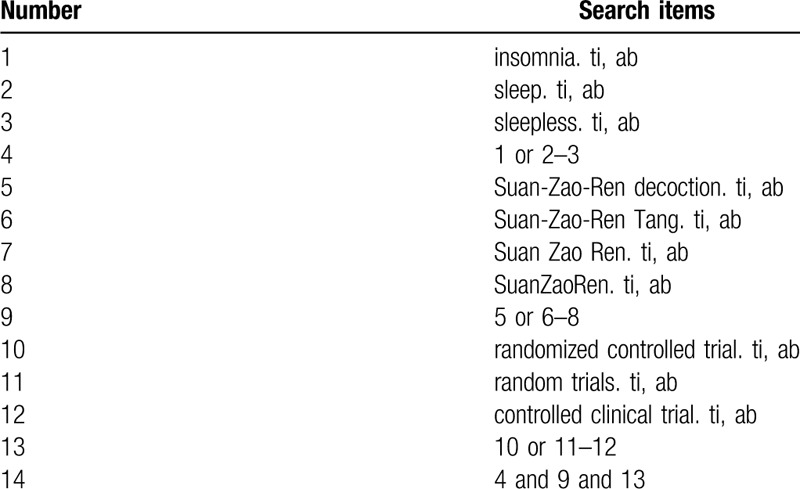
Search strategy used in PubMed database.

### 2.3 Data collection and analysis

#### 2.3.1 Selection of studies

**Figure 1:**
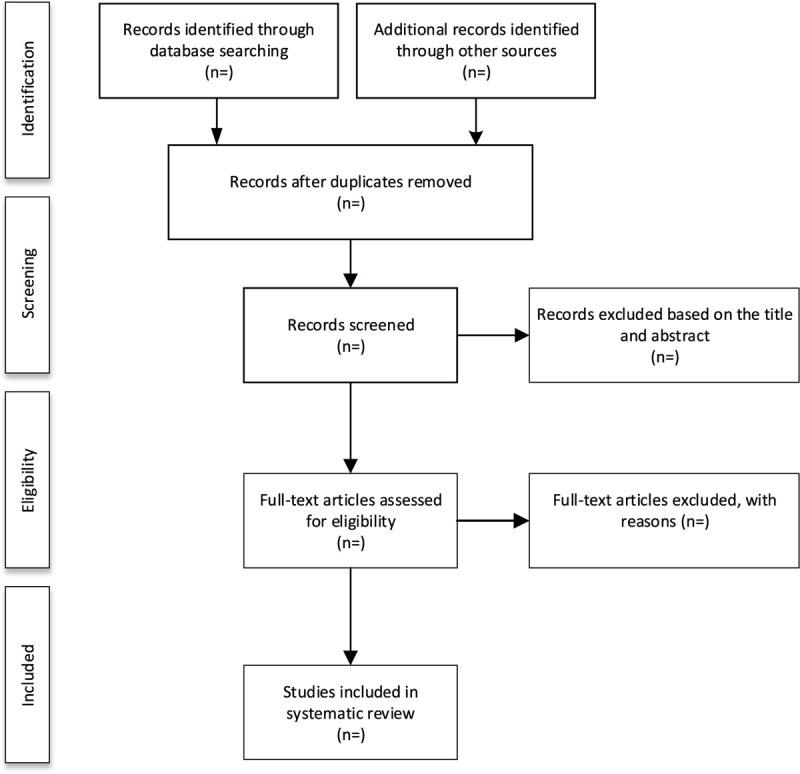
Flow diagram of study selection process.

We will import the data retrieved from the relevant database into EndNote X8 software. After that, two independent authors will read the title, abstract and full text of the literature according to the inclusion criteria to assess the eligibility of these articles. If there are any disagreements, the 2 authors will discuss and reach an agreement. The study selection procedure is summarized in Figure 1.

#### 2.3.2 Data extraction and management

Two authors will independently extract relevant data from the eligible RCTs, including author, participant characteristics, sample size, intervention details, follow-up, outcomes, and adverse events. The above information will be cross-checked by two authors. If necessary, we will also contact the corresponding author for more information.

### 2.4 Risk of bias assessment

We will evaluate the risk of bias from 7 items based on the Cochrane Collaboration's tool. The contents include: random sequence generation, allocation concealment, the blinding method for patients, researchers and outcomes assessors, incomplete result data, selective reports and other sources of bias. Each item will be rated as high, low or unclear risk of bias.^[21]^ The assessment will be completed by two authors independently, and all disagreements will be resolved through discussion.

### 2.5 Quantitative data synthesis and statistical methods

#### 2.5.1 Quantitative data synthesis

RevMan 5.3 software will be used for statistical analysis. Continuous results will be calculated as mean difference (MD) and 95% CI. Dichotomous outcomes will be calculated with the risk ratio (RR) and 95% CI.

#### 2.5.2 Assessment of heterogeneity

Heterogeneity will be evaluated by *I*^*2*^ test and Chi-square test. When *I*^*2*^ ≤ 50% and *P *> .10, the research result will be regarded as homogeneous; otherwise, it will be considered as heterogeneous.

#### 2.5.3 Assessment of reporting biases

We will examine the publication bias by evaluating the symmetry of the funnel plot. If the funnel plot is not symmetric, the results of the study may have a publication bias.

#### 2.5.4 Subgroup analysis

If there is significant heterogeneity in our study, we will perform a subgroup analysis based on the type of control group.

#### 2.5.5 Sensitivity analysis

If enough RCTs are included in our study, we will conduct sensitivity analysis based on study quality, sample size, and missing data to assess the robustness of the meta-analysis.

#### 2.5.6 Grading the quality of evidence

We will evaluate the quality of evidence by the Grading of Recommendations Assessment, Development and Evaluation and divide them into 4 levels: high, medium, low or very low.^[22,23]^

## 3 Discussion

Traditional Chinese medicine (TCM) has been widely used in the treatment of insomnia, with satisfactory therapeutic effects and fewer side effects. Among them, SZRD has always been popular for the treatment of insomnia and restless.^[24]^ Although the benefits of SZRD for insomnia have been widely reported,^[25,26]^ the effectiveness of SZRD has not been systematically and scientifically evaluated. The aim of this study is to evaluate the effectiveness and safety of SZRD for insomnia. The conclusion of this study may provide evidence-based medical advice for the treatment of insomnia by SZRD.

## Author contributions

**Data curation:** Zhijian Song, Qi Zhang.

**Formal analysis:** Zhijian Song, Yang Yang.

**Investigation:** Xueyu Liu, Qinan Zhan.

**Methodology:** Yurong Xiong, Yang Yang.

**Project administration:** Zhijian Song, Ping Fan.

**Software:** Qi Zhang, Qinan Zhan.

**Supervision:** Ping Fan.

**Validation:** Ping Fan, Xueyu Liu.

**Visualization:** Qi Zhang, Xueyu Liu.

**Writing – original draft:** Zhijian Song, Ping Fan.

**Writing – review & editing:** Zhijian Song, Ping Fan.
